# The impact of an educational pamphlet on the awareness of parents about 4‒6-year-old children’s oral habits and dentofacial discrepancies

**DOI:** 10.15171/joddd.2016.009

**Published:** 2016-03-16

**Authors:** Shahla Momeni Danaei, Fatemeh Faghihi, Ali Golkari, Maryam Saki

**Affiliations:** ^1^Professor, Department of Orthodontics, Orthodontics Research Center, School of Dentistry, Shiraz University of Medical Sciences, Shiraz, Iran; ^2^Student Research Committee, School of Dentistry, Shiraz University of Medical Sciences, Shiraz, Iran; ^3^Assistant Professor, Department of Community Dentistry, School of Dentistry, Shiraz University of Medical Sciences, Shiraz, Iran; ^4^Postgraduate Student, Department of Orthodontics, Orthodontics Research Center, Student Research Committee, School of Dentistry, Shiraz University of Medical Sciences, Shiraz, Iran

**Keywords:** Dentoskeletal discrepancy, oral habits, parental awareness

## Abstract

***Background. ***This study aimed to evaluate whether the parents’ knowledge about the adverse effects of oral habits and dentoskeletal discrepancies would improve by an educational pamphlet.

***Methods. ***A parallel-group randomized clinical trial was conducted on parents in kindergartens of Shiraz, Iran, 2013. The parents completed a designed questionnaire to determine the pre-intervention score. The study group received an educational pamphlet on the oral habits and dentoskeletal discrepancies, in contrast to the control group. Three weeks later, the parents in both groups took the questionnaire again (post-intervention score). The primary outcome was a change in the parents’ knowledge about oral habits and dentoskeletal discrepancies, which was measured by 13 questions of the questionnaire. Each correct answer was given a positive point and each incorrect answer a negative point. The total pre- and post-intervention scores were calculated by summing up the points and compared using Mann–Whitney U test.

***Results. ***A total of 550 subjects were assessed for eligibility and 413 were randomized. Of the study group, 203 subjects (98.56%), and of the control group, 204 parents (98.54%) completed the questionnaire for the second time. The score of the study group in the "normal occlusion" section of the questionnaire had significantly improved (P < 0.001) and in the "oral habits" section the score of both groups had improved but in the study group the improvement was significantly higher (P < 0.001).

***Conclusion***. The educational pamphlet can be effective in increasing the level of parents’ knowledge about normal occlusion and complications of oral habits.

## Introduction

Preservation of oral health cannot be achieved without raising public awareness.^1-4^ The importance of imparting knowledge in orthodontic treatment and taking charge of the oral health and treatment has been shown earlier.^5^ Malocclusion, as a worldwide health issue, has economic, psychological and social effects as well as functional and esthetic issues.^6-8^ Although malocclusion is the result of a combination of genetic or environmental influences, anthropological studies have shown that the primary etiology for changes noticed in the populations’ pattern of occlusion is environmental condition. Notable among these environmental conditions are oral habits which can be categorized into nutritive and non-nutritive.^9,10^ Non-nutritive sucking, such as the use of a pacifier, bottle-feeding and early weaning,^6,11,12^ is a common behavior among young children and its prevalence among different populations ranges from 0% to 46% in children.^10,13^ Persistent non-nutritive sucking of the thumb or other fingers leads to sagittal and irreversible discrepancies in dimensions of the maxilla and the mandible, depending on the intensity and the duration of the habit.^13^ Despite the fact that digit sucking and oral breathing habits and their adverse effects are usually detected, most parents often lack the appropriate motivation and knowledge to encounter the causes and fail to demand help from dentists when it is necessary.

The etiology, prevalence, adverse effects and management of digit-sucking habit in children have been studied in the literature. Little attention has been paid to the parents’ attitudes towards digit-sucking which is as an important point to consider in elimination of the habit.^10,14^ To improve public information about dentofacial discrepancies, we must first determine the present level of knowledge and sources of information.^14^ Helping individuals take responsibility for maintaining their oral health is a vital aim which cannot be served without public motivation and education. Studies have shown that written information can help patients understand and comply with their dentist’s or doctor’s advice. Pamphlets have the advantage of low cost and the patients do not have to ask embarrassing direct questions.^15^

Since continuing oral habits in the 4-6-year age group can cause dentofacial discrepancies, the aim of this study was to investigate the parental awareness (at an age range of 20-50) about dentofacial discrepancies and to assess the effect of an educational pamphlet on this issue in parents of 4-6-year-old children.

## Methods

### Ethical considerations

Ethical approval was received from the Committee of Ethics of Shiraz University of Medical Sciences. All the subjects were assured that they participated voluntarily, that they could quit the study at any time and that their data would be kept strictly confidential.

### Trial design and setting

A parallel-group randomized clinical trial was performed in kindergartens of Shiraz, Islamic Republic of Iran, from February to December 2013.

### Participants, recruitment and randomization

Parents (age range of 20-50) with one or more 4‒6-year-old children were identified and included in the study by one research assistant using registered data in kindergartens’ offices. A letter explaining the aim of the project and a consent form were sent to all the potential participants. A designed questionnaire was delivered to the parents with the help of their children. The validity of this questionnaire was assessed by submitting the questionnaire to one professor in each field of orthodontics, pedodontics and dental public health. The reliability of the questionnaire was assessed by asking 20 subjects to complete it twice with a 2-week interval. Cronbach’s α was used as a measure of reliability (α = 0.726). One schoolmaster called the parents on the registered available phone number and reminded to return the questionnaire in due course. The illiterate parents, those whose children were absent on the day of distributing the questionnaire, and those who did not complete the consent form, did not complete the questionnaire or did not return it on due course were excluded from the study.

The questionnaire had two parts and a total of 30 questions, consisting of “demographic” data and “parents’ knowledge” section. The “demographic” section included 12 questions about the name and gender of the child, name of the kindergarten, child’s birthplace and birth date, number of children in the family, occupation and education of parents, family income and a question to determine which parent filled the questionnaire. Parents who had more than one 4-6-year-old child were considered only once and the gender of the child who was responsible for the delivery of the questionnaire was recorded. The “knowledge” section consisted of two sets of questions:

A) Seven questions with more than one correct answer: two questions related to “child’s present and past oral habits”; three questions on “the best and easiest way of obtaining knowledge about dental and medical issues”; two questions about “complications of oral habits and dentoskeletal discrepancies” without treatment.

B) Eleven multiple choice questions with only one correct answer, in two major domains: seven questions for awareness about “normal occlusion and dentoskeletal relations” and four questions about “complication of oral habits”.

The instructions for answering were included in the questionnaire and no oral explanation was undertaken. After the completion of pre-intervention questionnaires, the parents were randomly divided into two groups: control and study. The sequence of randomization was carried out using a computerized random number generator and the allocation number was kept in sequentially numbered envelopes which are sealed and opaque. The randomization was independently conducted by a research assistant who was not involved in the investigation for eligibility, providing the pamphlet, or assessing the results.

### Intervention

Parents in the control group received an envelope containing an acknowledgement letter for participation in the study and encouragement to cooperate. The study group received an envelope in similar shape and color, in which both the educational pamphlet and the acknowledgement letter were enclosed. The envelopes were delivered by the children. On the first page of the pamphlet, an instruction was written for the parents to read the pamphlet over one week. No oral demonstration was performed. After one week, the parents in the study and control groups were expected to bring back their envelope with all of its containments. Again, they were reminded on the phone by one schoolmaster was accomplished.

The pamphlet was derived from one text book of orthodontics (Proffit WR, Sarver DM and Fields HW. *Contemporary Orthodontics*, 5th ed. St. Louis, MO: Elsevier Saunders; 2013) and pedodontics (Pinkham JR, Casamassimo PS, McTigue DJ, Fields HW, Nowak AJ, editors. *Paediatric Dentistry: Infancy through Adolescence*. 4th ed. St. Louis, MO: Elsevier Saunders; 2005) by one professor in each field of orthodontics and pedodontics and written in fluent Farsi language, comprehensible to lay people. The validity of the pamphlet was evaluated by asking two other professors of orthodontics and pedodontics. The pamphlet comprised of eight parts in question and answer form: “What are considered as oral habits?” “What is the ideal dento-skeletal condition in a 4-6-year-old child?” “What is the best approach to quit oral habits?” “What consequences can oral habits have?” “What causes mouth breathing?” “What problems can mouth breathing have?” “What are the signs of mouth breathing in a child?”

Two weeks after returning the envelope, the parents in both the control and study groups completed the questionnaire again to determine the post-intervention score. Once again, a telephone reminder was sent.

### Outcome measures

The primary outcome was expected to be a change in parents’ knowledge about oral habits, dentoskeletal discrepancies and their complications in a 4-6-year-old child. It was measured by 13 questions in the knowledge section consisting of seven questions on “normal occlusion” and six on “complications of oral habits”. Two of the questions about “complications of oral habits” are from the “set A” questions of the” knowledge part” with more than one correct answer. Each of these two questions had six alternative choices of which only three were correct. Their scores were measured so that each selected correct choice was given a positive point and each incorrect choice was given a zero. Therefore the maximum score which could be obtained from these two questions was six, with a minimum of zero. For the other 11 questions, from “set B” questions with only one correct choice, each correct answer was given a positive and each incorrect answer was given a negative point. The pre- and post-intervention total scores were calculated by summing up the points. Consequently, the maximum total score could add up to 17 (“normal occlusion”: 11, “complications of oral habit”: 6) and the minimal amount could be -11 (“normal occlusion”: -5, “complications of oral habit”: -6).

The secondary outcome was to determine the best way of obtaining dental information in the parents’ point of view. It was evaluated by the three questions regarding the best and easiest way of obtaining knowledge about dental and medical issues. The first round of completing the questionnaire was used for this purpose.

The tertiary outcome was to determine a crude prevalence of oral habits in 4-6-year-old children of Shiraz. It was assessed by the two questions of the questionnaire related to the child’s present and past oral habits.

### Sample size and data analysis

In Shiraz, primary education is divided into 4 districts within which people are roughly homogeneous socioeconomically. Overall, there were 1537 four-to-six-year-old children in 148 kindergartens distributed in these 4 districts. Since there was no similar study, sample volume was considered at least 400 with 200 subjects in each group, based on consultation with a statistician. Using stratified random sampling with proportionate allocation strategy, a total of 42 kindergartens were selected. The total score for each participant and the mean score for the two groups were calculated. Data were imported into SPSS software (SPSS Software, Version 13.0; LEAD Technologies, Inc., Chicago, IL). The pre- and post-intervention scores were compared using Mann–Whitney U test. The parents’ education and occupation, gender of the child, child’s birthplace, and family income, number of children and the person who completed the questionnaire were compared between the control and experimental groups using chi-squared test. The relation between these factors and the total score of participants were evaluated using ANCOVA. Parents’ opinion about the best way of gathering information on dental and medical issues was reported in percentage. The prevalence of oral habits was calculated both before 4‒6 years of age and in the present time in percentage.

## Results

**Figure 1.**
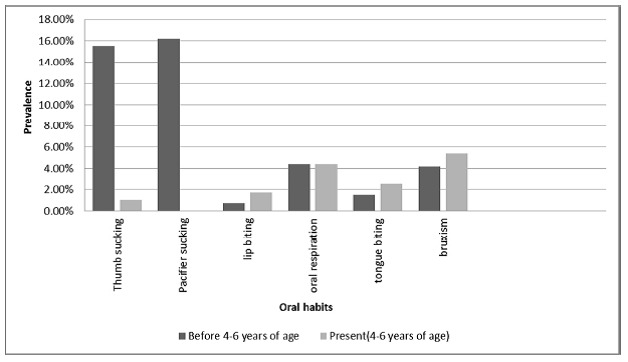
The flow of the participants through the trial.

Figure 1 shows the flow of the participants through the trial. Of the study group, 203 subjects (98.56%) participated in the second round of questionnaire filling while 204 parents (98.54%) in the control group completed the questionnaire for the second time. There was no significant relationship between the father’s job (P = 0.907, 0.843), mother’s job (P = 0.892, 0.458), mother’s education (P = 0.305, 0.393), the child’s birthplace (P = 0.438, 0.201), number of children (P = 0.843, 0.846), father's education (P = 0.857, 0.882), family income (P = 0.802, 0.614), and the pre- and post-intervention total scores of the parents, while the person completing the questionnaire (P = 0.502, 0.018) showed significant relationship with the post-intervention total score, with the gender of the child (P = 0.011, 0.401) demonstrating significant relationship with the pre-intervention total score.

### Primary outcome

The pre-intervention total score, pre-intervention score in the section of normal occlusion and the complications of oral habits were not different significantly between the two groups (P > 0.05). The mean post-intervention total score significantly improved in the study group (P < 0.001) in contrast to the control group (P = 0.616). Of the questions about normal occlusion, the score of the study group significantly improved at the post-test (P < 0.001) while the score of the control group did not change significantly (P = 0.176). Of the questions on complications of oral habits the score of both groups improved at the post-test (study group: P < 0.001, control group: P = 0.011) but the improvement was significantly higher in the study group (P < 0.001).

### Secondary outcome

The subjects declared that they obtained their medical and dental knowledge mostly by asking a medical doctor and a dentist (44.71% and 58.72%, respectively). Regarding the easiest way of obtaining information, 53.6% believed in reading books and brochures, 25% preferred television programs, 8.8% liked radio programs, 12.5% mentioned other sources such as searching the web, and asking friends or relatives.

### Tertiary outcome

**Figure 2.**
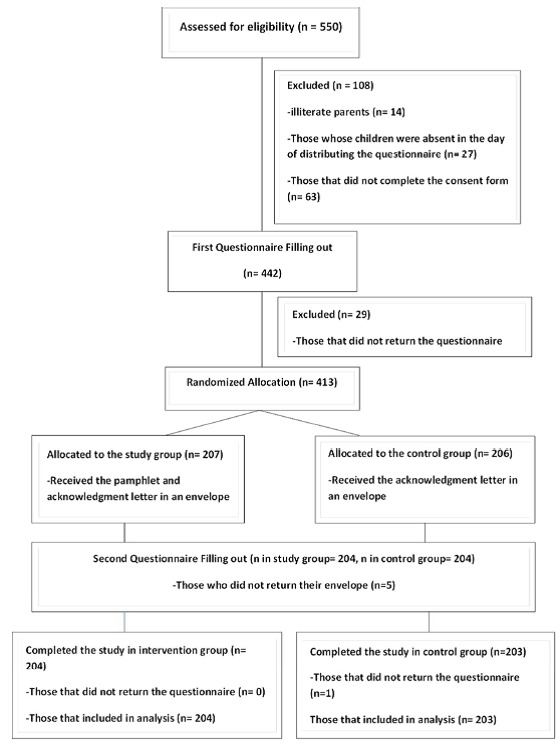
Prevalence of oral habits before 4-6 years of age and the present time reported by the parents.

Figure 2 illustrates the prevalence of oral habits before 4-6 years of age and the present time, reported by the parents. While thumb and pacifier sucking decreased, lip and tongue biting and bruxism increased. Mouth breathing did not exhibit any change. Prevalence of oral habits totally dropped from 38.6% to 12.5%.

## Discussion

In this study, 407 parents of 4-6-year-old children in kindergartens of different districts of Shiraz were selected to achieve socioeconomic diversity. There was no significant difference in the knowledge of parents about normal occlusion and complications of oral habits between the study and control groups before the intervention. Before giving the pamphlet, only the gender of the child was significantly related to the total score of parents. This confounding factor was controlled by ANCOVA which demonstrated no significant relation with the total score after the intervention. Furthermore, the study and control groups were matched regarding the gender of the child. After randomized allocation and the intervention, ANCOVA was performed once again. This time, the only significant factor that could affect the total score was shown to be the person completing the questionnaire. This confounding factor could not be controlled and was not matched between the two groups. More individual parents filled out the questionnaire in the control group while the number of the questionnaires that were filled by both parents was significantly higher in the study group (P = 0.007). Considering other factors, the proportion of highly educated fathers with non-official jobs in the study group was greater than that in the control group (P = 0.004 and P = 0.040, respectively). These two factors were not shown to affect the total score before and after the intervention. The two groups were similar regarding factors such as mother’s job and education, family income, number of children and birthplace. The knowledge about normal occlusion in the experiment group significantly improved and the awareness about complications of oral habits of both groups improved but the improvement was significantly higher in the pamphlet group.

In conclusion, factors such as parents’ education and job, family income, number of children and child’s birthplace did not affect parental awareness about dentofacial discrepancies while gender of the child could have an effect. This confounding factor was controlled so that it did not influence results. However, the factor of who filled out the questionnaire was not controllable and might have affected the results. Our findings are consistent with those of a Finnish study,^16^ which showed social class had no effect on needing or receiving treatment. However, according to Patel et al^17^ and Rud et al^18^, variations in the educational level of the participants might have an effect on the parents’ answers.

Improvements in the parental awareness about complications of oral habits of the control group might be attributed to the negative meaning implied by the word “complications”. Parents probably became more alert about the problems that might endanger their children and tried to carry out more investigations into the issue with other approaches they considered the best and easiest. For instance, in the current study, in relation to the easiest way of obtaining information, reading books and brochures, television programs, radio programs, other sources such as searching the web, and asking friends or relatives were determined as the preferable ways of obtaining information in the order of the most to the least. Besides, subjects declared that they obtained their medical and dental knowledge mostly by asking a medical doctor and a dentist. This factor was also uncontrollable.

The age range of children chosen for the current study was 4-6 years. This is the age that a constant oral habit can result in an abnormal development and growth of the craniofacial complex. At three years of age, oral habits, oral respiration, low tongue posture, and elongation of the lower anterior facial height are apparent but after age five they are more commonly noticed.^19^ Almonaitienė et al^20^ reported that oral habits could be found in 19.7% of children; the most common ones were nail biting and finger sucking, which accounted for 6% and7.9%, respectively. In our study, pacifier and thumb sucking were the most common behavior before 4-6 years of age while bruxism was the most prevalent oral habit in 4-6-year-old children. The prevalence of oral habits was estimated to be 12.5% among 4-6-year-old children in our research.

The subjects declared that they obtained their medical and dental knowledge mostly by asking a medical doctor and a dentist. The easiest way of obtaining information was shown to be reading books and brochures. The effectiveness of using a pamphlet might have resulted from its content and attractive format. Other studies showed that educational pamphlets can be effective for educating people. For example, a study was conducted in 2011 in Shiraz about the effect of an educational brochure on the parents’ awareness and knowledge of children’s orthodontic problems.^15^ In another study in 2013 in Shiraz the parents were given an educational leaflet about tooth avulsion.^21^ In addition, another study was performed to teach students aged 15‒18 about healthy sleep and showed the effectiveness of the designed pamphlet.^22^ Different studies have shown that written information could help patients understand and comply with the advice of their dentist or doctor. Fleckenstein’s brochure, given to every patient, had virtually 100% acceptance and cooperation. Weinman confirmed the value of pamphlet for patients, showing that 75% preferred written format of information and that 80% read the leaflets.^15^ Although leaflets have been found to be effective in raising awareness, they need to be suitably written to be comprehended.^23,24^ The effectiveness of suitable leaflets was confirmed by our study.

There were limitations to our study. The long-term holding of information was not evaluated. Factors such as the person completing the questionnaire and other sources of obtaining information for parents were uncontrollable. The section on presence of oral habits in children, before 4-6 years of age, was based on the parents’ recalling ability. The strengths of the study were that simple language was used in the leaflets and the questionnaire was formulated to be understood by a range of educational levels. To reduce bias, our researchers were trained not to give verbal information before and while the parents took part in the study and also the pamphlet group did not have the leaflet in the second round of answering the questions.

## Conclusion

The educational pamphlet can be effective in increasing the level of parents’ knowledge about normal occlusion and complication of oral habits.

## Acknowledgments

The authors thank Vice Chancellery of Shiraz University of Medical Sciences for supporting this research. The authors also thank Dr. Vosooghi of the Dental Research Development Center of the School of Dentistry for the statistical analysis and Dr. Nasrin Shokrpour at Center for Development of Clinical Research of Nemazee Hospital for editorial assistance. This article was derived from F. Faghihi’s thesis for the degree of Doctor of Dental Surgery.

## Authors’ contributions

The study was planned and designed by SMD, AG and FF. Literature search was carried out by all the four contributors. Data acquisition was conducted by FF and MS. Data interpretation was performed by FF and MS. Manuscript preparation was carried out by MS. Manuscript revision was carried out by SMD, AG and FF. Manuscript review was performed by MS. All the authors contributed to the final draft and approved the final manuscript.

## Funding

This work was supported and funded by Orthodontics Research Center of Shiraz Dental School with Grant #92-6349.

## Competing interests

The authors declare no competing interests with regards to authorship and/or publication of this article.

## Ethics approval

Ethical approval was received from the Committee of Ethics of Shiraz University of Medical Sciences. All the subjects were assured that they participated voluntarily, that they could quit the study at any time and that their data would be kept strictly confidential.
